# Effects of insecticidal proteins of *Enterobacter cloacae* NK on cellular immunity of *Galleria mellonella* larvae

**DOI:** 10.3389/fmicb.2023.1154811

**Published:** 2023-05-09

**Authors:** Chunli Liao, Ran Huang, Yi Yang, Yapeng Huang, Kai Zhang, Liang Ma, Taotao Li, Lianzhe Wang, Huamin Zhang, Bingbing Li

**Affiliations:** ^1^College of Life Science and Engineering, Henan University of Urban Construction, Pingdingshan, Henan, China; ^2^Center of Healthy Food Engineering and Technology of Henan, Henan University of Urban Construction, Pingdingshan, Henan, China; ^3^Laboratory of Water Pollution Control and Rehabilitation Technology, Henan University of Urban Construction, Pingdingshan, Henan, China

**Keywords:** insecticidal protein, *Galleria mellonella*, cellular immunity, inhibition, *Enterobacter cloaca*

## Abstract

*Enterobacter cloacae* produces insecticidal proteins capable of causing toxicity in pests, but the insecticidal mechanisms of these proteins for insect control remain unclear. To elucidate the mechanisms, the purified insecticidal protein from *E. cloacae* NK was administered to *Galleria mellonella* larvae either by intraperitoneal injection or by feeding. The number of hemocytes, apoptosis in immune cells, and polyphenol oxidase (PO) activity of *G. mellonella* larvae were detected by hemocytometer, Annexin V-FITC/PI, and UV–vis spectrophotometer, respectively. With the extension of the invasion time of NK insecticidal protein, the number of hemocytes in *G. mellonella* larvae decreased significantly (*p* < 0.05), whereas the apoptosis rate of hemocytes increased. The activity of PO showed a trend of rising-peak-sharp decline and the melanization reaction was deepened simultaneously. Moreover, the phagocytosis and coating capabilities of hemocytes decreased, and the intraperitoneal injection method was more effective than the feeding method. Taking together, the insecticidal protein of *E. cloacae* NK inhibits and destroys the cellular immune response of *G. mellonella* larvae, which suggests an important role in killing the host insect.

## Introduction

Insects survive well for hundreds of millions years on the earth mainly due to their robust innate immune system formed during the long-term evolution (Liu and Yuan, 2018). When insects are infected by parasites (parasitic wasps, pathogenic microorganisms, etc.) or threatened by the surrounding environment, a strong natural immune system will be activated (Awori, 2022), protecting them from adverse influences (Lemaitre and Hoffmann, 2007). The immune system of insects consists of cellular immunity and humoral immunity (Tsakas and Marmaras, 2010). Cellular immunity mainly involves hemocytes in the hemolymph, which is an important constitution of the immune system (Zhang et al., 2018; Li et al., 2020). Cellular immunity plays a very important role in insect defense against external pathogens through recognition by the relevant receptors, which then triggers the insect’s immune defense response. Importantly, both immune responses are not absolutely independent but interconnected, promoted, and coordinated (Shi et al., 2012).

The nematophilic symbionts have mainly been found to be the three genera *Xenorhabdus*, *Photorhabdus*, and *Serratia nematodiphila* (Zhang et al., 2008, 2009, 2012). However, our recent study reported that *Enterobacter cloacae* NK is a novel symbiotic bacterium of *Heterorhabditis* in the gut of *G. mellonella*, suggesting *Enterobacter* as another ematophilic genus (Liao et al., 2017). After the nematodes enter the insect body cavity, they release the symbiotic bacteria in their intestines (Lara-Reyes et al., 2021). In recent years, researchers have found that nematophilic symbionts were used in the research of human diseases. The symbiotic relationship between entomopathogenic nematodes and host insects can be employed as a new model to study some complex human diseases, such as neurodegenerative diseases and cancer (Markaki and Tavernarakis, 2010; Tissenbaum, 2015; Denzel et al., 2019). In addition, understanding the pathogenicity of entomopathogenic bacteria can help to study some similarities of human pathogens, and the discovery of this orthologous virulence pathway could reveal strategies to prevent and treat human *Drosophila kiwi* infection (Swart et al., 2022).

Secondary metabolites, such as protein toxins produced by nematophilic symbionts, were used as biopesticides or drugs to solve the problems of public health and agriculture caused by insects and fungal pathogens (Cimen et al., 2022). However, it is necessary to have an in-depth understanding of the action mechanism before formally application. At present, the insecticidal proteins of *E. cloacae* include Tc protein, Xpt protein, Txp protein, Mcf protein, and so on (Bowen and Ensign, 1998; Morgan et al., 2001; Daborn et al., 2002). These insecticidal proteins elicit immune responses in insects by destroying host immune cells and inhibiting phagocytosis of hemocytes (Shi et al., 2013), resulting in apoptosis and cytolysis (Park et al., 2007), or by inhibiting the activation of polyphenol oxidase (Anju et al., 2015) and the transcription of the bacterium peptide gene. Our previous studies have found that the insecticidal protein of *E. cloacae* has a strong destructive effect on the midgut tissue of *G. mellonella* larvae (Liao et al., 2021), probably by inducing insect humoral immune response (Liao et al., 2019). However, there are few studies on the cellular immunity of insecticidal proteins produced by *Enterobacter* spp.

In this study, we attempt to examine the effects of insecticidal proteins of *E. cloacae* NK on cellular immunity of *Galleria mellonella* larvae. With different interferences of insecticidal proteins, the number of hemocytes, apoptosis in immune cells, and polyphenol oxidase (PO) activity of *G. mellonella* larvae are used for detection of responsive actions from the larvae to unveil how cellular immunity mechanisms takes place. Our findings will provide an important foundation for further clarifying the insecticidal mechanism by studying the cellular immunity of this insecticidal protein.

## Materials and methods

### Purification and extraction of insecticidal proteins

**SCHEME 1:**
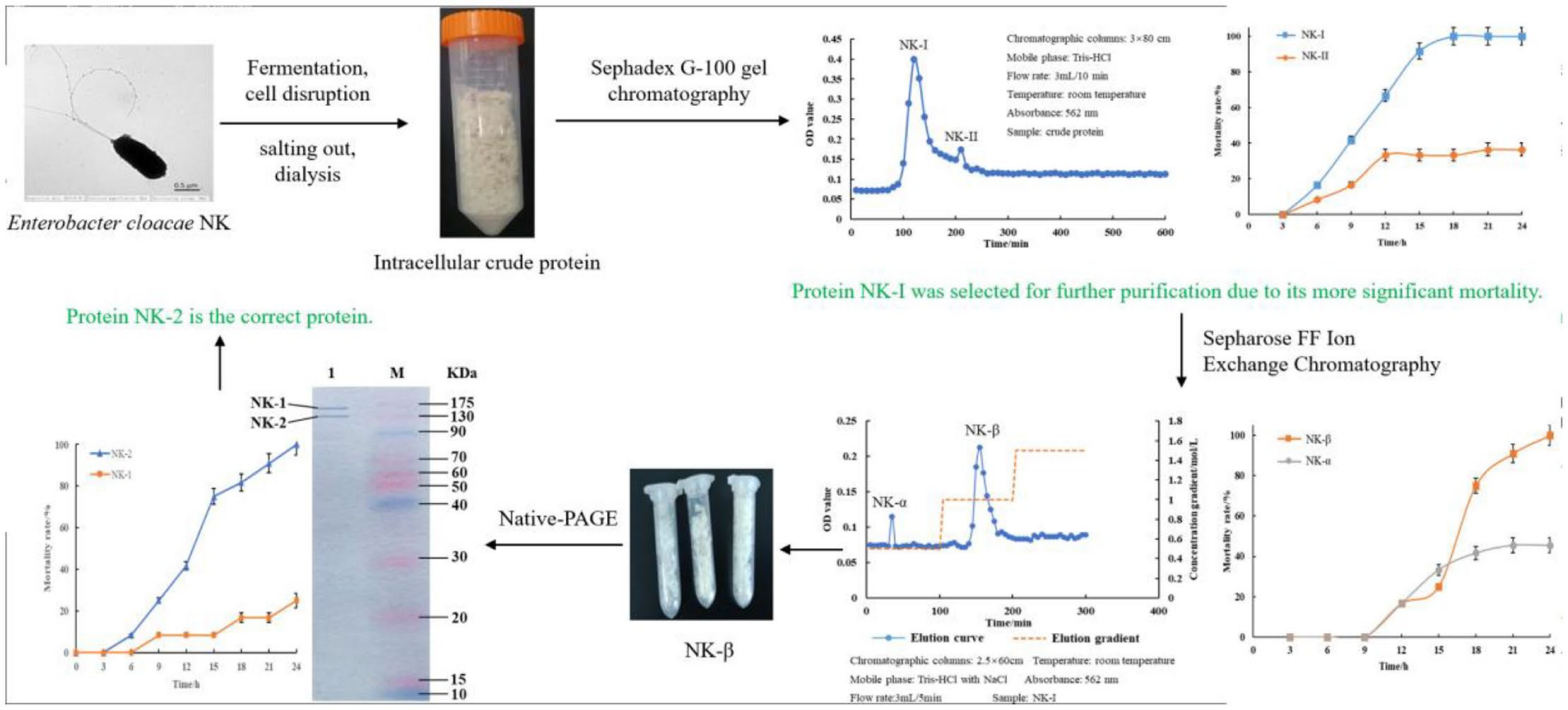
Workflow to purify the bacterial protein.

After fermentation of *E. cloacae* NK (Peptone 2.1%, Glucose 0.6%, MgSO_4_ 0.13%, Na_2_SO_4_ 0.15%, (NH_4_)_2_SO_4_ 0.23%, KH_2_PO_4_ 0.072%, K_2_HPO_4_ 0.098%, ph 7.2–7.4), bacterial cells were collected, sonicated, salted out, and dialyzed (Liao et al., 2017). Finally, the purified protein was obtained through Sephadex G-100 gel chromatography and DEAE-Sepharose FF ion exchange chromatography (−80°C storage). The strategy to purify the bacterial protein was performed as Scheme 1.

### Culturation of experimental insects

Experimental group 1: one hundred and twenty larvae of *G. mellonella* were selected and divided into three groups. Larvae were injected with the insecticidal protein solution (0.2 mg/ml) of *E. cloacae* NK and cultured at 22°C for 8 h. Then, 10 larvae were randomly sampled every 2 h from each group for collection of hemolymphs and hemocytes.

Experimental group 2: one hundred and twenty larvae were selected and divided into 3 groups. The larvae were fed with the food (honey 25%, glycerol 20%, flour 50%, yeast 5%) mixed with *E. cloacae* NK insecticidal protein (0.2 mg/g) for 4 days. Thereafter, 10 larvae were randomly sampled from each group every 1 day to collect hemolymph and hemocytes.

Control group: the control group 1 was injected with normal saline, whereas the control group 2 was fed with the mixed food of normal saline. The culture conditions were the same as above.

### Hemocyte collection

The larvae of the control groups and the experimental groups were collected at the different times, and their body surfaces were disinfected with 75% alcohol. Their tails were cut off and the hemolymph was immediately dropped into a 1.5 ml EP tube. Then, 20 μl of hemolymph were transferred into 180 μl of precooling anticoagulant for 5 min centrifugation (2000 *g*, at 4°C), and the hemocyte pellets were collected for further experiments.

### The variation of hemocyte counts

The hemocyte pellets collected were first transferred into a 1.5 ml EP tube and then diluted to 10^−6^–10^−7^ cell/mL using sterile PBS. Thereafter, 1 ml of the suspension was added into 200 μl of the Medium Grace’s insect cell liquid. After gently mixed, 10 μl of cell suspension were loaded onto the glass slide for counting. The variation of hemocyte counts was observed and calculated on the hemocyte counting plate under the phase contrast microscope, following the formula below:

Cell density (cell/mL) = (Sum of cells in the 80 wells/80) × 400 × 10^4^ × Dilution fold.

### Detection the apoptosis of hemocytes

According to the specification of the Annexin V-FITC/PI Apoptosis Detection Kit, the hemocyte cell suspension was diluted by 1 × Binding buffer to adjust the hemocyte concentration within 5–10 × 10^5^/mL. Then, 200 μl of the cell dilution were transferred into a 1.5 ml EP tube and mixed slowly with 5 μl of the Annexin V-FITC at room temperature in dark. After 10 min, 5 μl of PI were added for 5 min incubation before supplementing with PBS to 500 μl. Finally, the apoptosis of hemocytes was detected by the flow cytometer (MILLIPORE 6-2 l) within 1 h, where 10,000 cells per sample were collected and the results were analyzed by the Flowjo V 10 Software.

### Detection the activity of polyphenol oxidase

Detection of the PO activity was referred to the method by Gauillard et al. (1993). Briefly, a total of 20 μl hemolymph was mixed well with 1 ml PBS containing 1 μg laminarin and 0.01 mmol/l L-dopa. Absorbance at 495 nm was measured by UV spectrophotometer (Thermo Fisher 10S UV–VIS). The activity was defined as the variation in the absorbance value within 5 min.

### Effects on hemocyte coating

The hemocyte concentration was adjusted to 1 × 10^6^/ml. Then, 20 μl of the hemocyte suspension were loaded into each well of a 96-well plate, supplementing with 180 μl of Medium Grace’s insect cell liquid and 1 μl of the Sephadex G-100. Finally, the plate was incubated at 25°C for 6 h to observe the coating of cells under a phase contrast microscope.

### Effects on cytophagy

The larvae of the control group and the experimental group were injected with 5 μl of the Green fluorescent microspheres. Hemocytes were collected after 2 h. Phagocytosis of the Green fluorescent microspheres by hemocytes was observed under a fluorescent inverted microscope with brightfield and 580 nm excitation light, respectively. Phagocytosis occurred when the cells contained the fluorescent signals, while the rest were in a free state. The operation was repeated three times to calculate the phagocytosis rate.

### Statistical analysis

All the data were generated from the three biological replicates. Data were expressed as mean ± SD in each chart. Differences between samples were examined using the Student’s *t*-test or two-way ANOVA. Statistically significant difference was evaluated at *p* < 0.05.

## Results

### Effects of insecticidal protein NK on the number of hemocytes in *Galleria mellonella* larvae

**Figure 1:**
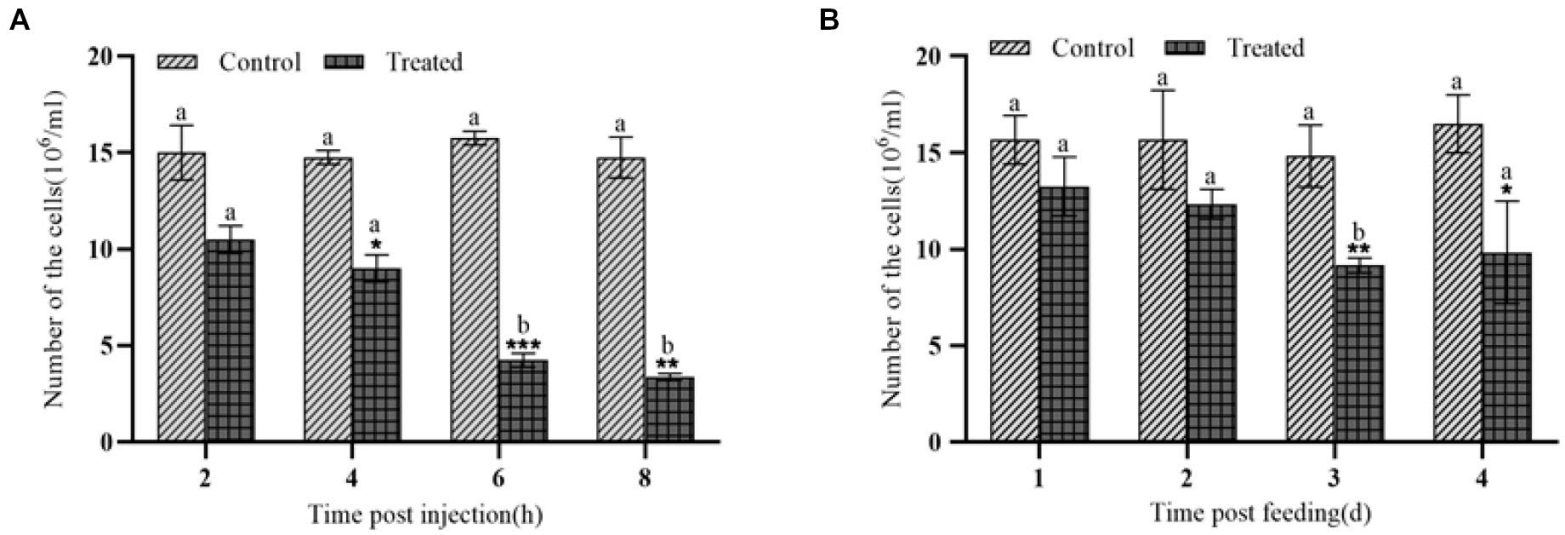
Changes in the number of hemocytes after NK insecticidal protein invaded *G. mellonella* larvae. **(A)** Changes in the number of hemocytes after injection of NK insecticidal protein; **(B)** changes in the number of hemocytes after feeding with NK insecticidal protein. ‘*’ was the significant difference between the control group and the experimental group of NK insecticidal proteins, a, b, and c represent the significant difference between different time periods (*t* test, *p* < 0.05), and the subsequent significance analysis is carried out in the same way. *P < 0.05, **P < 0.01.

*Galleria mellonella* was treated by intraperitoneal injection of body cavity or by feeding with the insecticidal protein of *E. cloacae* NK, and its effects on the number of hemocytes in *G. mellonella* larvae was detected. Compared with the control, both injection and feeding with insecticidal protein NK resulted in a significant decrease of the number of hemocytes in *G. mellonella* larvae (Figures 1A,B). Importantly, the injection is more effective compared to feeding. Specifically, after 8 h injection of the insecticidal protein, the number of hemocytes in *G. mellonella* larvae was only 22.88% of that of the control group; however, after feeding the insecticidal protein for 4 days, the number of hemocytes was still 59.57% of that of the control. Our results showed that insecticidal protein NK can effectively decrease the number of immune hemocytes, which might finally cause the death of *G. mellonella* larvae.

### Effects of insecticidal protein NK on apoptosis of hemocytes of *Galleria mellonella* larvae

**Figure 2:**
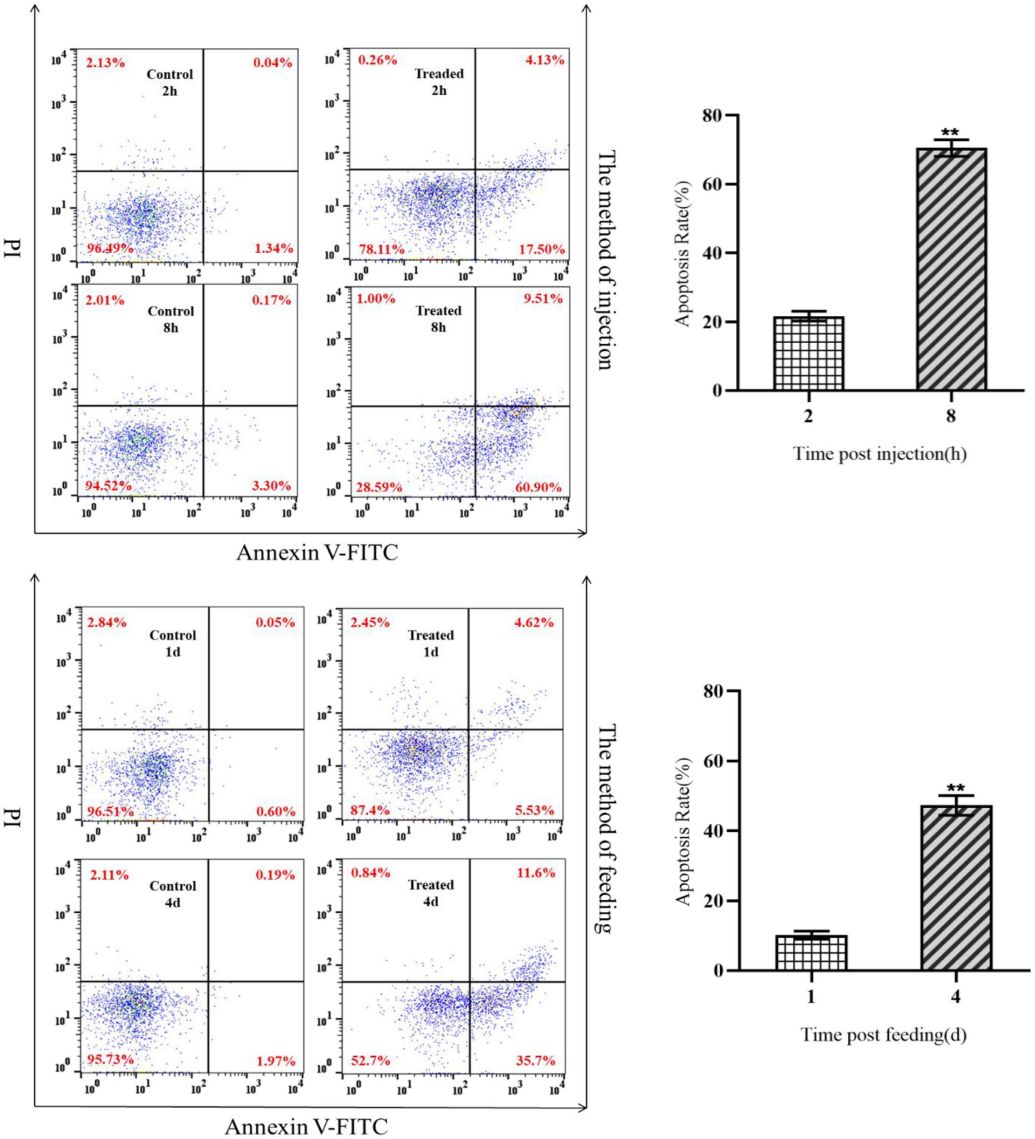
Apoptosis of hemocytes after NK protein invaded *G. mellonella* larvae.

The effects of insecticidal protein NK on immune cell apoptosis in *G. mellonella* larvae were compared at different times. Moreover, the relationship between the change of the number and apoptosis of hemocytes was analyzed. As a result, the early/late apoptosis rate of hemocytes increased from 21.63 to 70.41% by intraperitoneal injection of body cavity for 8 h, while that of hemocytes in the control group unchanged (Figure 2). After 1 day and 4 days feeding with insecticidal protein NK, the early/late apoptosis rate of hemocytes increased from 10.15 to 47.30%, but the apoptosis rate of hemocytes in the control unchanged (Figure 2). Both intraperitoneal injection and feeding with insecticidal protein NK, the hemocytes had obvious apoptosis, however, the rate of apoptosis in the feeding method was relatively lower than the former. Therefore, it is speculated that the invasion of insecticidal protein NK would cause apoptosis of hemocytes, which results in a decrease in the number of hemocytes and further decline in the cellular immunity of the host insect, leading to the death of *G. mellonella* larvae eventually.

### Effects of insecticidal protein NK on polyphenol oxidase activity and melanization reaction in the hemolymph of *Galleria mellonella*

**Figure 3:**
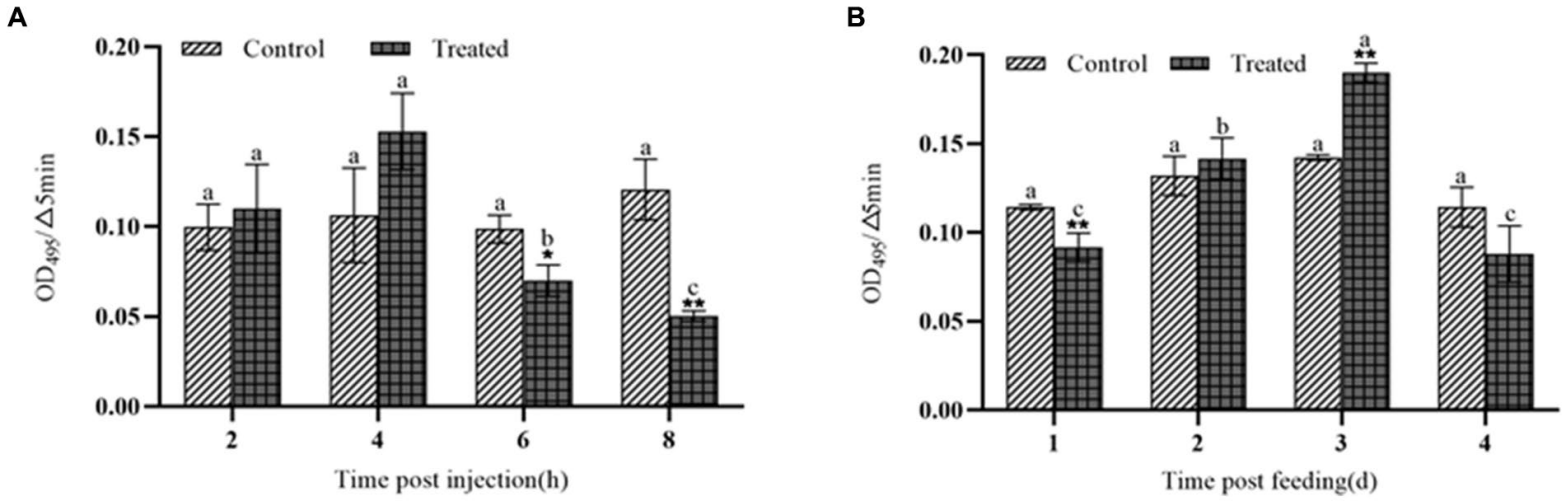
The effect of NK protein on the activity of polyphenol oxidase in the hemolymph of *G. mellonella* larvae. **(A)** Changes in the activity of polyphenol oxidase by injection NK protein; **(B)** changes in the activity of polyphenol oxidase after feeding NK protein. *P < 0.05, **P < 0.01. a, b, and c represent significant differences (*p* < 0.05) between different time periods.

**Figure 4:**
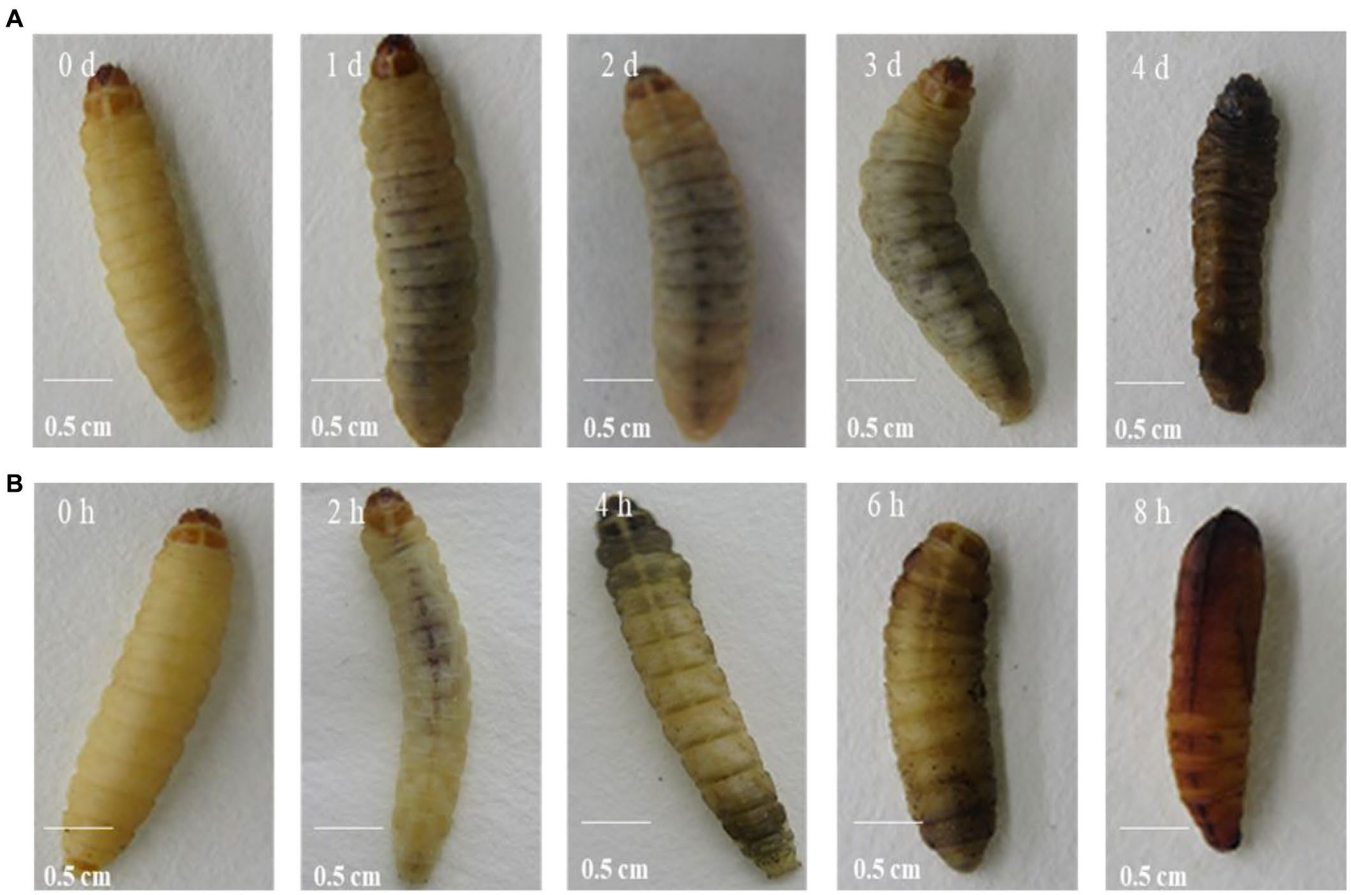
NK protein induced melanization of *G. mellonella* larvae through feeding **(A)** and direct injection **(B)**.

After injection of the protein NK, the PO activity increased significantly within 4 h, peaked at 4 h, and then decreased sharply after 4 h (Figure 3A). After feeding with the protein NK, the PO activity showed the same trend as the injected protein, but this change needed 4 days (Figure 3B). The melanization reaction of *G. mellonella* larvae was caused by activation of polyphenol oxidase (Figure 4). Both intraperitoneal injection and feeding with insecticidal protein NK showed that the PO activity increased in the early stage, indicating that the PO cascade reaction might be activated to resist the function of the insecticidal protein. However, the PO activity decreased sharply when the time prolonged. We speculated that the insecticidal protein NK might inhibit the PO activity and damage the host immune system. In addition, the long-term melanization reaction could produce toxic substances that attack the pathogenic substances and the insects themselves, which may be an important reason for the death of *G. mellonella* larvae.

### Effects of insecticidal protein NK on the hemocyte coating of *Galleria mellonella* larvae

**Figure 5:**
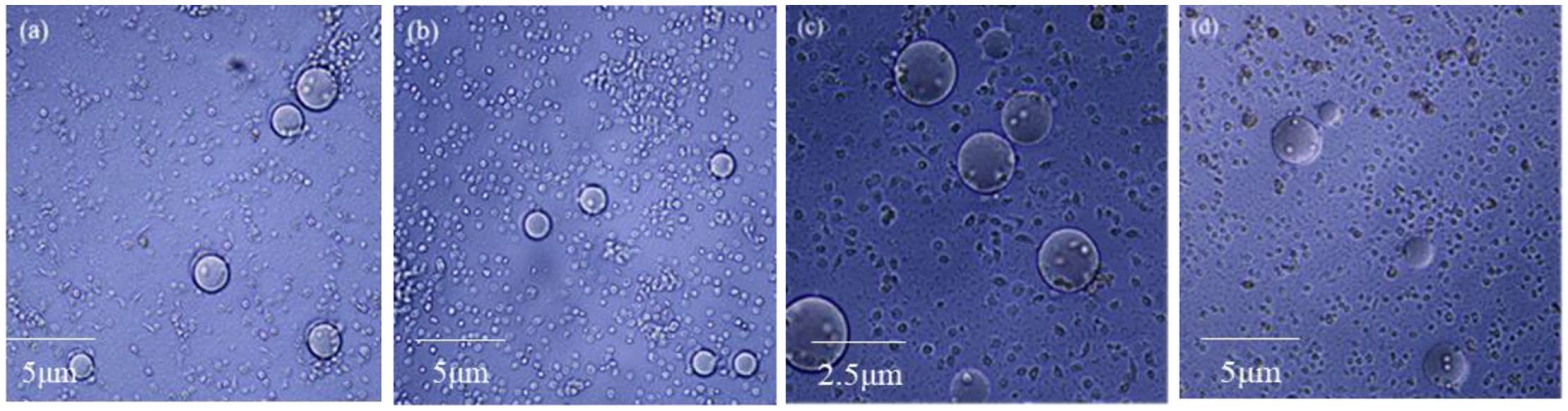
The effect of NK insecticidal protein on the coating ability of *G. mellonella* larvae hemocytes. **(A)** Untreated 8 h hemocyte coating; **(B)** treated of injection 8 h hemocyte coating; **(C)** untreated 4 days hemocyte coating; **(D)** treated of feeding 4 days hemocyte coating.

The coating ability of *G. mellonella* larvae hemocytes was detected by agarose beads. The more hemocytes wrapped around the agarose beads, the stronger the coating capacity is. After 8 h injection and 4 d feeding with insecticidal protein NK, the hemocytes were collected to observe the coating activity on agarose pellets. Both injection and feeding treatments significantly inhibited the coating ability of hemocytes, and the number of hemocytes aggregated in spheroids was also reduced (Figure 5). When the coating ability of hemocytes declines, the cellular immune function of *G. mellonella* larvae will also reduce. Thus, the cellular immune system might not resist the invasion of insecticidal proteins, leading to death eventually.

### Effects of insecticidal protein NK on the phagocytosis of phagocytose

**Figure 6:**
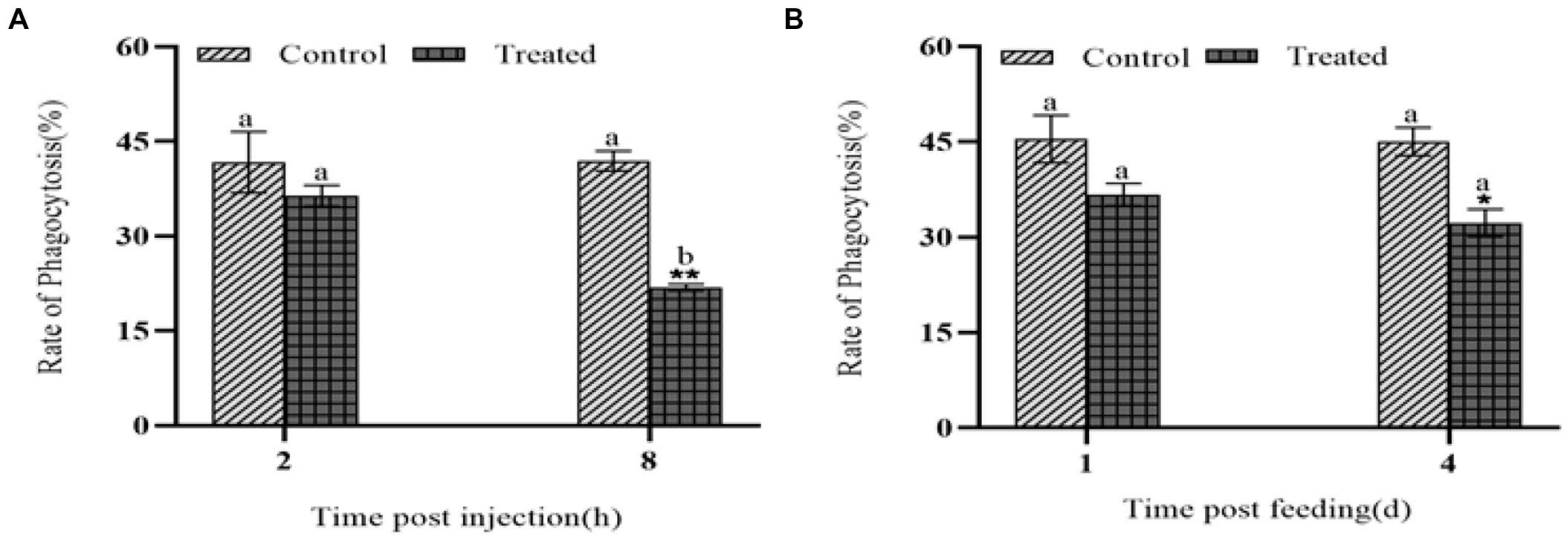
The effect of NK protein on the phagocytic ability of hemocyte. **(A)** Changes in phagocytic ability of hemocytes after injection of NK insecticidal protein; **(B)** changes in phagocytic ability of hemocytes after feeding NK insecticidal protein. *P < 0.05, **P < 0.01. a, b, and c represent significant differences (*p* < 0.05) between different time periods.

When foreign substances invade the hemolymph of *G. mellonell* larvae, the hemocytes in the lymph can phagocytose them. After injection of the protein NK, the phagocytic ability of *G. mellonella* larvae hemocytes decreased (Figure 6A). At 8 h, the phagocytic rate of *G. mellonella* larvae was only 21.8%, which was much lower than 41.9% of the control group, showing that the phagocytic ability of hemocytes significantly decreased. After feeding with insecticidal protein NK, the phagocytic ability of hemocytes also decreased, compared with the control, but the decrease was smaller than that of the injection treatment (Figure 6B).

## Discussion

Hemocytes in insect lymph fluid play an important role in various physiological processes of insects, such as metabolism, metamorphosis and innate immunity (Zhang et al., 2018). In this study, when the protein NK was injected or fed, the number of *G. mellonella* larvae hemocytes decreased significantly. In the apoptosis experiment, the apoptosis rate of hemocytes reached the similar level (55.5 and 39.56%, respectively) after injection and feeding of the protein. It shows that apoptosis might be an important reason for the decrease of the hemocyte number. Therefore, it is speculated that insecticidal protein NK could induce apoptosis of the hemocytes of *G. mellonella* larvae in a certain way, resulting in their death. Some studies showed that after the injection of *XeGroEL*, an insecticidal protein of *Pathogen cloacae*, the hemocytes of *G. mellonella* larvae sharply decreased shortly, instead of apoptosis (Shi et al., 2013). Although insecticidal proteins can reduce the number of hemocytes in *G. mellonella* larvae, they have different mechanisms, implying the diversity of the toxic mechanism of the insecticidal proteins.

Most insects can trigger a cascade reaction of polyphenol oxidase after being invaded by pathogens, which results in the melanization reaction within a few minutes (Matsumoto et al., 2022). This melanization reaction produces some toxic small molecular substances that can help kill most pathogens (Ashida et al., 1990). In this study, after injection and feeding of the protein NK, PO activity in *G. mellonella* larvae was significantly activated, compared with the control group. This suggests a protective mechanism of insects against the invasion of foreign substances. After PO is activated, some black toxic substances are produced in the insects to wrap the invading pathogenic substances or to kill them through the melanization reaction on the body surface of the insects (Ozakman and Eleftherianos, 2021). However, with the prolongation of the reaction time, the activity of PO began to decrease, probably because the immune system of *G. mellonella* larvae was severely damaged. On the other hand, the long-term melanization reaction produced a large amount of toxic substances, which might attack the pathogenic substances and the insects themselves. These may be one of the important reasons for the final death of the *G. mellonella* larvae. It was found that after the entomopathogenic nematodes invaded *G. mellonella* larvae, the PO activity of *G. mellonella* larvae was inhibited and its melanization reaction was correspondingly decreased (Yang et al., 2012). In contrast, other studies also found that PO levels remained unchanged in *Drosophila melanogaster* larvae infected with *H. gerrardi*, compared to uninfected *D. melanogaster* larvae (Lara-Reyes et al., 2021). From this point of view, although entomopathogenic nematodes and symbionts co-invade insects in a parasitic relationship, their pathogenic mechanisms to insects are different.

Certain hemocytes of insects are phagocytic and immune, which can help insects resist the invasion of external pathogens (Rizki and Rizki, 1984; Gray and Botelho, 2017). We found that the phagocytosis and coating ability of *G. mellonella* larvae hemocytes decreased after treatment of the protein NK. PO not only mediates humoral immunity, but also can mediate cellular immunity, such as phagocytosis and coating (Liu et al., 2013). It is reported that after injection of PO antibody, the phagocytosis of *E. coli* to hemocytes was significantly blocked, showing that the PO cascade is involved in the phagocytosis process (Mavrouli et al., 2005). When the *DmPPO1* and *DmPPO2* genes of *Drosophila* were knocked out, the ability of *Drosophila* to resist the invasion of pathogenic substances was significantly reduced, compared with the wild type, which eventually led to a shortened lifespan (Binggeli et al., 2014). In this study, whether the decreased phagocytosis and coating ability of *G. mellonella* larvae cells are affected by PO activity needs further verification.

Taking together, the insecticidal protein produced by *E. cloacae* NK may directly attack hemocytes and cause apoptosis after entering the body cavity of *G. mellonella* larvae, resulting in the inhibition of PO activity and the sharp decline in phagocytosis and coating ability. In conclusion, the protein NK might inhibit the immune response of *G. mellonella* larvae and play an important role in the interaction between symbiotic bacteria and host insects. As the immune response is basic, further work is required to unveil how this insecticidal protein interferes with antimicrobial peptide production and immunological priming.

## Data availability statement

The raw data supporting the conclusions of this article will be made available by the authors, without undue reservation.

## Author contributions

CL and BL: conceptualization, supervision, and funding acquisition. CL: methodology, software, validation, formal analysis, writing – original draft preparation, visualization, project administration, and resources. CL, RH, YY, KZ, TL, LW, and YH: investigation. CL, RH, and BL: data curation. CL, RH, YY, LM, HZ, and BL: writing – review and editing. All authors contributed to the article and approved the submitted version.

## Funding

This study was supported by the Henan Province Science and Technology Research Project (grant no. 222102110001) and the Henan Province Key R&D Special Project (grant 221111110900).

## Conflict of interest

The authors declare that the research was conducted in the absence of any commercial or financial relationships that could be construed as a potential conflict of interest.

## Publisher’s note

All claims expressed in this article are solely those of the authors and do not necessarily represent those of their affiliated organizations, or those of the publisher, the editors and the reviewers. Any product that may be evaluated in this article, or claim that may be made by its manufacturer, is not guaranteed or endorsed by the publisher.
